# Autism's Impact on Cochlear Implantation Surgery Outcomes in Deaf Children

**DOI:** 10.22038/IJORL.2024.74369.3502

**Published:** 2024-05

**Authors:** Masoud Naderpour, Yalda Jabbari Moghaddam, Amin Abbasi, Aida Ariafar, Bita Poorshiri

**Affiliations:** 1 ***Department of Otorhinolaryngology, Faculty of Medicine, Tabriz University of Medical Science, Tabriz, Iran. ***; 2 ***Research Center for EvidenceBased Medicine, Iranian EBM Centre: A Joanna Briggs Institute Affiliated Group, Tabriz University of Medical Sciences, Tabriz, Iran.***; 3 *Department of Pediatrics, Tabriz University of Medical Sciences, Tabriz, Iran.*

**Keywords:** Autism spectrum disorder, Cochlear implantation, Hearing loss, Speech ability.

## Abstract

**Introduction:**

Cochlear implants (CI) provide a hearing sense for severe to profound hearing-impaired patients, both adults and children, and they are a broadly effective and accepted therapeutic method for those patients. Also, Deaf children with comorbidities, including autism spectrum disorders (ASDs), undergo cochlear implantation. ASDs are a group of developing disorders characterized by abnormalities in social interaction and communication with limited repetitive patterns of behavior. This study aimed to assess the effect of Autism on CI surgery outcomes in Deaf Children.

**Materials and Methods:**

We followed 12 autistic patients with cochlear implantation and 12 non-autistic cochlear-implanted patients for two years. The Categories of Auditory Performance (CAP) and Speech Intelligibility Rating (SIR) scores were used to assess 6, 12, and 24 months after cochlear implantation surgery.

**Results:**

During the 24-month follow-up, the CAP means scores increased in both groups, and SIR and CAP progresses were considerably greater in non-ASD children (P<0.001). However, in ASD children, the progress of CAP and SIR variables were significant, with 99% and 95% confidence, respectively, at 24 months after surgery.

**Conclusion:**

Although the CIs could improve hearing performance in autistic patients, speech development after CIs in autistic children could affected by several factors, including the severity of autism, and this can be effective in providing pre-implant counseling to parents. The application of the alternative communication methods could be taken into account as a potential rehab technique.

## Introduction

Autism is a neurodevelopmental illness that usually affects the early years of life. Autism Spectrum Disorders (ASDs) or Pervasive Developmental Disorders (PDDs) are a group of disorders that include autism, Asperger's disorder, and Pervasive Developmental Disorder-Not Otherwise Specified (PDD-NOS). Three main components of ASDs are communication difficulties, impaired social interaction, and limited behaviors and interests (1). Although symptoms of autism usually appear by age 3, the majority of children are not diagnosed until age four (2). The prevalence of ASD is 1000/6, and it is about four times higher in males than females (3). In Iran, the prevalence of typical autism is 6.26 per 10,000, which is about one-tenth of the world (4). 

Because developmental biological markers are unavailable, accurate diagnosis of childhood developmental disorders is based completely on behavioral criteria. Diagnosis is especially challenging in children with global developmental delay, in whom two or more of these conditions co-exist, so it is hard to recognize hearing impairment and autism in a child with other disabilities. However, parents frequently notice developmental difficulties before the first year of life, with concerns of vision and hearing most reported in that first year (5). Jure et al. stated that in a deaf children’s school, 4% of children met the criteria for an autism diagnosis (6). Gillberg et al. discovered that 30% of children with autism had severe to moderate conductive hearing deficits (7). In other research, found that 21% of patients with autism had sensorineural hearing loss (8). Only extensive studies can determine the causal relationship between autism and deafness. In Iran, Daneshi and Hassanzade reported additional disabilities in 15% of a total of 60 cochlear-implanted, prelingually deaf patients and 6.66% of them had autism (9). 

The diagnosis of hearing loss and ASD is crucial as approximately 40% of children with a known long-lasting hearing problem have another disability (10). There is a wide range of ways in which hearing is affected by autism. For many, the nerves transporting sound to the brain might fail (known as auditory processing disorder, APD), and the children find it hard to understand what they hear. On the other hand, the most noticeable clinical features of hearing problems in individuals with ASD are abnormally increased sensitivity to loudness and behavioral reactions triggered by environmental sounds (11). In ASD children, the most common sensory impairment is the auditory hyper or hypo sensitivity that interrupts behavioral adaptation (12). Magnetoencephalography (MEG) studies also showed that auditory stimuli were associated with delayed responses in autism (13). Matsuzaki et al. stated that auditory hypersensitivity in ASD is a specific reaction of the primary auditory cortex, probably due to functional abnormalities or neurological immaturity of the cortex (14). Cochlear Implantation (CI) is one of the most effective interventions in profound to severe sensorineural hearing loss (SNHL). It facilitates the sound introduction for young patients with severe to profound hearing impairment. SNHL is a complicated disorder with intense cultural, social, and medical consequences. The measure of hearing is decibels (dB) and severity of hearing loss is graded as profound (more than 90 dB), severe (71–90 dB), moderately severe (56–70 dB), moderate (41-55dB), or mild (26–40 dB) (15,16). Cochlear implant systems consist of a microprocessor programmed and an externally worn microphone to extract timing, frequency, and intensity cues from acoustic signals. These acoustic cues are then transformed into electrical codes by the system. Internally, in the cochlea, a receiver placed by surgery relays the transmitted code to an implanted array of contacts to stimulate surviving auditory neurons. A child’s response to a cochlear implant can be affected by Cognitive disorders, and children with cognitive disorders, such as autism or intellectual disabilities, might improve partially, sound responsiveness only, or even no improvement (17). Only a few published studies have discussed the effect of ASD on CI outcomes on deaf children's performance. It is challenging for clinicians to assess children diagnosed with ASD for cochlear implant indication and post-operative programming such patients. Our study's novelty lies in its focus on elucidating the specific impact of ASD on cochlear implantation outcomes in children with bilateral deafness, helping clinicians develop more personalized treatment plans based on each patient's unique needs and circumstances and providing valuable insights into delivering safe and effective care to all patients regardless of their health status.

## Materials and Methods

In this prospective cohort study, we enrolled 24 patients who underwent cochlear implantation at the Cochlear Implant Center of Tabriz in Iran between 2011 and 2018. Patients were divided into two groups based on the presence or absence of ASD: the ASD group (n=12) and the non-ASD group (n=12). 

Inclusion criteria were patients between 3 to 7 years old with confirmed ASD diagnosis by a team consisting of a neurologist, a psychologist, and a speech therapist using diagnostic criteria according to the DSM-5 and who had deafness or severe hearing loss and were undergoing cochlear implantation. Exclusion criteria encompassed patients with normal hearing or mild hearing loss, and those with any accompanying problems related to hearing loss, such as physical or other mental conditions. 

Additionally, patients not meeting the specified age range or not enrolled at the designated center during the specified timeframe were excluded from the study. ASD was diagnosed before implantation in 5 children and after implantation in 7 children. The severity and demographic data of our study are provided in Table 1. 

**Table 2:** Demographics and ASD severity of patients

Subject.no	Sex	Severity of ASD
**1**	female	severe
**2**	male	severe
**3**	male	severe
**4**	male	severe
**5**	female	severe
**6**	female	mild
**7**	male	mild
**8**	male	mild
**9**	male	moderate
**10**	female	moderate
**11**	male	moderate
**12**	male	moderate

All subjects had bilateral severe to profound hearing loss as determined by preoperative audiometry with and without a hearing aid and an ABR test. Cochlear implant devices from two manufacturers, Med-El (Med-El Company, Innsbruck, Austria) and Nucleus (Cochlear Ltd., NSW, Australia), were used for the implants. The choice of device in cochlear implantation did not impact the study results.

A non-randomized allocation approach was used to ensure the comparability of the two groups. 

Patients in the non-ASD group were matched to patients in the ASD group based on important variables that may impact surgical outcomes, such as age and severity of hearing loss. 

The non-ASD group was randomly selected from congenital deaf children in the same age range who underwent cochlear implantation at the same center and received auditory verbal therapy and special sensory therapy. The study followed all patients prospectively for two years after cochlear implantation to assess surgical outcomes.

To evaluate the outcomes of cochlear implantation, we assessed patients 6, 12, and 24 months after implantation using two reliable and valid instruments: the Speech Intelligibility Rating (SIR) and the Children's Auditory Performance Scale (CAP) II. Both scales are commonly used in Iran to assess speech production and auditory perception of cochlear implant recipients (18). 

The SIR scale, provided in, assessed the children’s speech intelligibility after cochlear implantation by evaluating their spontaneous speech. The scale has five categories ranging from "pre-recognizable words in spoken language" to "connected speech is understandable to all listeners." The scale allows for objective evaluation of speech fluency and intelligibility.

**Table 3:** Speech Intelligibility Rating (SIR) criteria Category

1. pre-recognizable words in spoken language (the child’s primary mode of everyday communication may be manual).
2. Connected speech is meaningless; understandable speech develops in single words when context and lip-reading cues are available.
3. Connected speech is understandable to listeners who concentrate and lip-read within a known context.
4. Connected speech is understandable to a listener with little experience of a deaf person’s speech; the listener does not need to concentrate excessively.
5. Connected speech is understandable to all listeners; the child is understood easily in everyday contexts.

In addition to the SIR scale, we used the CAP II to assess the auditory perception abilities of the children after cochlear implantation. The scale evaluates the child's ability to detect, discriminate, and identify sounds in different listening conditions. Categories of Auditory Performance II (CAPII) is a 9-point scale ranging from use of the telephone with a known speaker to no awareness of environmental sounds. CAPII is used to assess the perception of sounds in Cochlear-implanted children (Table 4).

**Table 5:** Categories of Auditory Performance II

**Category**	**CAP**
0	No alertness of surrounding sounds
1	Environmental sounds alertness
2	Response to speaking sounds (e.g., go)
3	perception of surrounding sounds
4	Discrimination of speaking sounds
5	Recognize common phrases, no lip-reading
6	Recognize conversation, no lip-reading
7	Use of telephone — known speakers
8	Use of telephone — unknown speakers

A speech therapist administered SIR and CAP II tests, blinded to the patient’s group allocation and pre- and post-operative test results. Test scores were recorded and analyzed to evaluate the outcomes of cochlear implantation. We conducted our statistical analysis using SPSS version 22 software, adhering to a significance criterion of p < 0.05. We employed the ANOVA repeated measure test to compare the progress of non-ASD and ASD children at 6, 12, and 24 months after surgery. Additionally, post-hoc analyses were performed to examine further the statistical significance of the comparisons between the two groups at each time point.

***Ethical considerations***

This study adhered to the principles outlined in the Declaration of Helsinki and was approved by the Tabriz University Committee on Ethics in Medical Sciences Research (ethical code: IR.TBZMED.REC.1398.052). All patients or their legal guardians were given a clear explanation of the study's objectives and

procedures, as well as information on potential benefits and risks. Participants provided written consent for participation and were assured that their data would be kept confidential. They were also informed of their right to withdraw from the study at any time. Throughout the investigation, patient data was kept confidential.

## Results

The results of the ANOVA repeated measure indicated a significant difference between the non-ASD and ASD groups during the different periods after surgery in both SIR and CAP variables (p<0.001). The mean data shows more progress in the group of non-ASD children. Additionally, the study of auditory progress with CAPII reveals that mild ASD results are closer to those of non-ASD children. Post-hoc analyses revealed that the comparison between the two groups in these three follow-up time points was statistically significant (p<0.001) (Fig 1).

**Fig 2:**
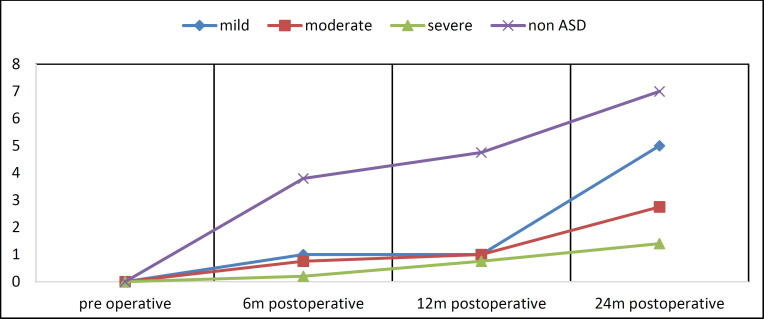
Comparison based on severity of ASD

The Kruskal-Wallis test was used to compare the difference between different months in children with ASD, and the same method was used for non-ASD children. The results show that in children with ASD, the progression during different months was significant (in the CAP variable) with 99% confidence, and according to the average rankings, the highest progress was observed in the 24th month, followed by the 12th month. The lowest amount of progress was observed in the sixth month after surgery. In the SIR variable, the progress at 24 months was significantly greater than at 12 months, and at 12 months was greater than at six months after surgery with 95% confidence (p<0.45). In non-ASD children, there was a significant difference between different months in both CAP and SIR variables with 99% confidence (p<0.001).

## Discussion

Our statistical analysis suggests that cochlear implantation can lead to progress in children with ASD and severe to profound deafness, particularly in the area of hearing. The improved CAP scores indicate that all children could perceive speech and environmental sounds after implantation. These findings are consistent with the results of Lachowska et al., who found that cochlear implantation is an effective treatment method for patients with severe to profound hearing impairment (18).

Donaldson et al. also supported the positive effects of cochlear implantation on ASD children, with improvements observed in features closely associated with ASD (19). Thompson and Yoshinaga Itano demonstrated that cochlear implantation can have a significant impact on children with in-born intensive bilateral hearing loss and ASD diagnosed in the early elementary school period. Despite their parents' knowledge of sign language, these children often had difficulty replicating signs, such as facial expressions and hand gestures, and their progress in sign language was slow. However, cochlear implantation dramatically improved their ability to listen and speak. Although social language skills may still be challenging for those with implants, they can communicate through speech.

Numerous studies have shown that cochlear implantation in autistic children does not typically lead to the development of language and speech, even after several years. In our experience, the outcomes of cochlear implantation in children with ASD are highly variable, and progress in language, speech, and hearing abilities varies depending on the severity of ASD. Out of our group of 12 ASD children, four have developed functional spoken language, while three of them did not use the prosthesis after two years despite its ability to create hearing. This issue is due to hypersensitivity in ASD children, who often demonstrate abnormal auditory processing or apparent hypersensitivity to sound (20). 

However, other parents in our study reported positive benefits of cochlear implantation, such as changes in behavior and communication, increased awareness of the environment, improved response to sound and music, increased eye contact, vocalization, use of sign language, and response to requests. These improvements were observed in five cases, consistent with the findings of Donaldson et al. (19). Our experience has taught us that since ASD affects each child differently, it is crucial to understand how the disorder may impact their ability to hear and process sounds. ASD children often present with tactile, visual, and auditory perceptual disorders, such as hyper- or hypo-sensitivity. Therefore, in-depth research into cochlear implantation outcomes in children with autism by specialized hearing and neurology teams can effectively develop theories and find auditory maps of these children. One such theory is the Weak Central Coherence (WCC) theory, which suggests that people with ASD have difficulty integrating data into a meaningful whole while their ability to process detailed information is improved or preserved (21). Several theories and hypotheses attempt to explain the autistic pattern of auditory symptoms. These theories lead to more effective therapies and more accurate predictions, helping professionals and parents anticipate improvement in the months and years following implantation. Our study demonstrated that cochlear implants have a positive prognosis in ASD children, which can aid parents and professionals in pre-implant consultations and candidate selection. Additionally, our speech results suggest that oral communication may not be the primary goal for implanted children with ASD, and other communication approaches, in addition to early diagnosis and intervention, may yield better results.

## Conclusion

In conclusion, cochlear implantation in children with ASD can lead to positive outcomes in hearing, speech, and communication abilities, although the results are highly variable, and progress may depend on the severity of ASD. While cochlear implantation may not lead to language and speech development in all cases, other communication approaches and early intervention can improve outcomes. Further research into the impact of ASD on hearing and auditory processing, as well as specialized pre-implant counseling, can help parents and professionals in candidate selection and setting realistic expectations for outcomes.

## Declaration:

*Consent for publication: *Not applicable 

### Ethics approval:

The medical ethics committee of Tabriz University of Medical Science approved this study with ethics ID: IR. TBZMED. REC. 1398. 052. This research was carried out following the Declaration of Helsinki.

### Availability of data and Materials:

This published article and its supplementary information files contain all the data generated or analyzed during the study.

### Funding:

We acknowledge that this project was conducted without external funding.

### Conflict of interests:

The authors declare that there is no conflict of interest regarding the publication of this paper.
